# The benefits of coronavirus suppression: A cost-benefit analysis of the response to the first wave of COVID-19 in the United States

**DOI:** 10.1371/journal.pone.0252729

**Published:** 2021-06-03

**Authors:** James Broughel, Michael Kotrous

**Affiliations:** Mercatus Center at George Mason University, Arlington, Virginia, United States of America; University of Waterloo, CANADA

## Abstract

This paper estimates the benefits and costs of state suppression policies to “bend the curve” during the initial outbreak of COVID-19 in the United States. We employ an approach that values benefits and costs in terms of additions or subtractions to total production. Relative to a baseline in which only the infected and at-risk populations mitigate the spread of coronavirus, we estimate that total benefits of suppression policies to economic output are between $632.5 billion and $765.0 billion from early March 2020 to August 1, 2020. Relative to private mitigation, output lost due to suppression policies is estimated to be between $214.2 billion and $331.5 billion. The cost estimate is based on the duration of nonessential business closures and stay-at-home orders, which were enforced between 42 and 65 days. Our results indicate that the net benefits of suppression policies to slow the spread of COVID-19 are positive and may be substantial. We discuss uncertainty surrounding several parameters and employ alternative methods for valuing mortality benefits, which also suggest that suppression measures had positive net benefits.

## Introduction

During the spring and summer months of 2020, many U.S. states enforced non-pharmaceutical interventions (NPIs) that sought to suppress COVID-19 transmission among the general population, namely by closing nonessential businesses and enforcing stay-at-home orders for all residents. According to the Institute for Health Metrics and Evaluation (IHME), between April 4, 2020 and April 24, 2020, 38 U.S. states and the District of Columbia actively enforced “stay-at-home” orders for their residents [1]. During this time, almost 90 percent of the total U.S. population was required to stay at home unless engaged in “essential” activities. These policies, and the pandemic generally, had substantial impacts on economic output and production, causing a recession in the United States.

In this paper, we offer a cost-benefit analysis (CBA) for the legal orders implemented across the United States to address the first wave of the coronavirus. We calculate the impacts of suppression policies on economic output and total production from March 2020 up to the week ending August 1, 2020. On the cost side, this includes losses to output associated with the enforcement of nonessential business closure and stay-at-home orders, as well as any indirect costs stemming from increased mortality risks associated with lost income. On the benefits side, we value prevented COVID-19 deaths in terms of total production gained by extending lives. We also consider the economic cost-savings associated with preventing COVID-19 illnesses and health-care utilization. Following this approach, we estimate that total benefits of state suppression policies are between $632.5 billion and $765.0 billion during the first wave of the coronavirus in the United States. The costs of suppression policies are estimated to be between $214.2 billion and $331.5 billion. Our results indicate that the net benefits of suppression policies to slow the spread of COVID-19 are positive and may be substantial.

This paper proceeds as follows. The second section describes the methodology for this CBA. The third section presents our calculations of benefits, costs, and net benefits. Our analysis focuses on economic production. However, given disagreement surrounding competing approaches to valuing the benefits of life-saving public health interventions during the COVID-19 pandemic, we also present estimates of mortality benefits using the value of a statistical life (VSL), as well as quality-adjusted life years (QALYs) saved. The fourth section discusses the key contributions of this article, as well as limitations of our analysis and remaining areas of uncertainty. The fifth and final section concludes.

## Methods

We estimate the net benefits of U.S. state policies to slow the spread of COVID-19 in terms of their estimated effects on economic output and total production. To calculate total benefits, we compare the observed impacts of the COVID-19 pandemic in the United States between early March 2020 and August 1, 2020 (a period during which “suppression” policies were enforced by most U.S. states) against a counterfactual scenario in which only targeted “mitigation” was practiced during that time span. Suppression policies aim to reduce viral transmission among the general population and keep case numbers low, and most U.S. states enforced some version of these policies for at least several weeks between March and August 2020. During this time, many state governors declared states of emergency and issued public health directives that required residents to stay-at-home and for public schools, higher educational facilities, and nonessential businesses to close. Between March 17, 2020 and August 1, 2020, 39 states (including Washington, D.C.) enacted stay-at-home orders, and 35 states required all nonessential businesses to close for a period of time. As of April 4, 2020, all 50 states and Washington, D.C. had closed educational facilities. S2 Appendix shows a full list of state policies and their dates of enforcement, as reported by IHME [1].

Meanwhile, mitigation strategies seek to reduce the health impact of an epidemic by reducing the exposure of at-risk populations [2]. We use Ferguson et al.’s forecast under their “most effective” mitigation strategies as our counterfactual progression of the COVID-19 disease from March to August 2020 [2]. Under this mitigation scenario, Ferguson et al. estimate that the United States would see “a single, relatively short epidemic,” in which 1.1 to 1.2 million deaths would occur, almost all of them before August 2020 [2]. In that model, most of the sick isolate, other members of their households voluntarily quarantine, and elderly individuals and other high-risk populations practice social distancing behaviors. The authors assume that a significant share (though not all) of the affected population voluntarily comply with case isolation and household quarantines for three months, roughly from April through June 2020, while elderly individuals maintain social distancing for a fourth month (July 2020) as well [2]. While the authors assume that social distancing of elderly individuals will be ordered by governments, we believe it is reasonable to assume that elderly and other high-risk populations would engage in social distancing behaviors even without government enforcement. Accordingly, this scenario reflects what may have happened during the first wave of COVID-19 cases if state governments across the United States had allowed private businesses and individuals to respond to the coronavirus pandemic as they saw fit, instead of enforcing COVID-19 suppression policies during these months, as they did.

Perhaps the most consequential benefit of government coronavirus suppression orders is preventing deaths from COVID-19. We value prevented COVID-19 deaths according to individuals’ expected remaining contributions to societal production (what we call a “value-of-production” approach). This approach distinguishes our study from some other analyses of the benefits and costs of slowing the spread of COVID-19 that use the VSL [3, 4]. Unlike our approach, the VSL incorporates non-pecuniary benefits such as “leisure, time with friends and family, and consumption of goods and services” [4].

Our approach to valuing mortality reductions is also distinct from human capital approaches to valuing life, which emphasize the present value of future earnings. Both the human capital and production values of life measure the marginal contributions individuals make during their lifetimes, and both measures generally decline with age. However, the human capital approach has been the subject of criticism [5, 6]. One limitation is that it often fails to account for the influence of contributions that take place outside of market activity, for example in the home. In other words, earnings do not account for the full spectrum of ways people contribute directly or indirectly to output and national income. By contrast, the approach taken in this article accounts for both market and nonmarket production. A further distinction is that, with the human capital approach, the value of life gradually depreciates to zero by the end of life [7]. Production value, meanwhile, aims to account for externalities generated by passed-on wealth after death [8, 9]. In theory, the production approach could value a death as a benefit when one’s remaining lifetime production is negative. This could be the case for a small minority of the population who are unable to work or to contribute to household production, perhaps due to illness or age. The lives of such people have intrinsic value to their family, friends, and society more broadly. Nevertheless, a production metric must account for the fact that resources that are consumed have an opportunity cost. An important opportunity cost of lost production is the alternative use of resources to address other mortality risks [10], which is an opportunity cost explicity accounted for in this paper.

When calculating benefits of suppression measures, we consider other factors that have incremental effects on productive output. Our approach to calculating benefits is “bottom-up” in the sense that we aggregate estimates of symptomatic infections, hospitalizations—including intensive care unit (ICU) stays and mechanical ventilation—and lung damage under suppression policies as well as under the baseline scenario of private mitigation. We do not estimate an additional impact of suppression policies on the future path of gross domestic product (GDP), since most of the factors included in our benefits estimate should contribute to GDP, and we seek to avoid double-counting. To stay consistent with our “value-of-production” approach to valuing mortality benefits, we value the benefits of prevented illnesses and health-care utilization in terms of the cost of service, rather than in terms of willingness to pay to avoid infection or to accept sickness in exchange for monetary compensation.

When estimating the health benefits of suppressing COVID-19, our analysis focuses only on the impacts of COVID-19 on adults, specifically people ages 18 and older. The U.S. Centers for Disease Control and Prevention (CDC) reports that, as of August 1, 2020, 98.7 percent of COVID-19 hospitalizations in its COVID-NET Network of hospitals were for adults 18 years or older [11, 12]. Meanwhile, the total number of COVID-19 deaths observed among children younger than 15 years old was approximately 0.04 percent of all COVID-19 deaths as of August 1, 2020 [13]. The fact that so few children have died of COVID-19 makes inferences about the impact of COVID-19 on children in the U.S. subject to substantial uncertainty. For this reason, we focus our benefits estimates on U.S. adults.

To calculate total costs of coronavirus suppression, we multiply the incremental daily costs to output of enforcing suppression policies by the duration, measured in days, that policies were enforced between March 2020 and August 1, 2020. To maintain consistency with our benefits estimates, our analysis of costs focuses on the incremental cost of suppression policies relative to the baseline scenario of private mitigation described by Ferguson et al. [2]. We also consider the indirect effect that forgone income may have on mortality, as described by Broughel and Viscusi [10]. Once all calculations have been conducted, we aggregate cost and benefit estimates in order to produce an estimate of the net benefits of suppression policies.

Because alternative approaches to valuing mortality benefits have been used in prior literature and because significant uncertainty and disagreement exist about the proper valuation metric to employ for mortality benefits during the COVID-19 pandemic, we provide supplemental benefits estimates using the VSL and QALY metrics. These estimates are not directly comparable to other benefits and costs calculated in this study, which are not valued on the basis of the current population’s willingness to pay for the benefit or to avoid the cost, or in terms of QALYs saved or lost. Nevertheless, we believe presentation of these alternative benefit estimates can be useful for highlighting some of the uncertainty that arises in CBA of policies that affect mortality. In this case, however, the sign of our net benefits calculation remains positive even when these alternative value-of-life metrics are employed. We discuss other areas of remaining uncertainty surrounding key parameters driving our conclusions in the discussion section.

## Results

### Benefits analysis

#### Reduced mortality

The CDC reports that 160,802 people ages 15 and older had died of COVID-19 in the U.S. up to the week ending August 1, 2020 [13]. The CDC does not report the number of deaths specific to ages 18 to 24, so we use the total number of deaths for ages 15 to 24 to represent that age group [13]. (Given the small number of deaths among those 15 to 24 years old, the resulting overcount is small.) Meanwhile, Ferguson et al. estimate that the United States would see 1.1 to 1.2 million deaths before August 2020 under the “most effective” mitigation strategy [2]. That model assumes that the infection fatality rate (IFR) increases significantly with age, so most of these deaths would be among adults [2]. Comparing observed deaths in the U.S. against the counterfactual mitigation scenario, we estimate between 940,000 and 1.04 million deaths among U.S. adults were prevented by the enforcement of state suppression policies.

To put a monetary value on the benefit of each life saved, we use the present value of workers’ remaining lifetime production. Our estimates of lifetime production come from Grosse et al. and are adjusted for inflation and productivity growth since 2007 (see S1.1 Table in S1 Appendix) [14]. That study calculated the present value of total worker production, including nonmarket production such as household production, for the American population, broken down by age group. Because the study includes nonmarket production, it is unlikely to discriminate against those who, for example, choose to stay at home to raise children rather than seek employment. Accounting for nonmarket production is desirable because replacing workers within a household or other nonmarket sector will affect market production. For example, if a grandparent who spent time at home cooking, cleaning, or rearing grandchildren dies, resources might have to be reallocated from market sectors to replace these household services. Moreover, because Grosse et al. include a detailed breakdown of production value by age [14], their analysis provides a more precise estimate of the value of reduced mortality than does the common practice of relying on population-average values of life.

According to the estimates in Grosse et al., expected lifetime production varies substantially with age, with prime-working-age people having higher expected lifetime production remaining than elderly and very young individuals, when a 5 percent discount rate is applied [14]. Accordingly, we compute a weighted average of lifetime production according to the age distribution of COVID-19 deaths in the United States, shown in Table 1. We calculate an expected benefit of approximately $338,000 in lifetime production per life saved from death by COVID-19.

**Table 1:** Expected lifetime production lost to COVID-19 deaths.

Age	Present value of lifetime production, 2020 USD	Number of COVID-19 deaths	Approx. share of COVID-19 deaths	Expected lifetime production lost
18 to 24	$1,700,684	298	0.2%	$3,152
25 to 34	$1,743,368	1,317	0.8%	$14,279
35 to 44	$1,511,338	3,326	2.1%	$31,260
45 to 54	$1,102,485	8,622	5.4%	$59,114
55 to 64	$626,928	20,378	12.7%	$79,449
65 to 74	$305,058	34,272	21.3%	$65,017
75 to 84	$163,013	42,143	26.2%	$42,722
85 plus	$137,889	50,446	31.4%	$43,258
All	—	160,802	100.0%	**$338,251**

*Sources*: [13, 14]; authors’ calculations.

*Note*: Sums may not be exact owing to rounding.

To calculate the value of mortality benefits, we simply multiply our estimate of the expected benefits from each prevented COVID-19 death by the projected number of prevented deaths. Multiplying $338,000 by the range of estimates of lives saved—940,000 on the low end and approximately 1.04 million on the high end—yields a gross estimate of $317.7 billion to $351.5 billion in benefits from reductions in mortality alone. To be clear, this is a gross estimate of the benefits of prevented mortality. In the cost analysis below, we estimate costs associated with any countervailing increases in mortality risk owing to the effects of depressed economic activity and income loss.

#### Reduced illness, health-care utilization, hospitalizations, and ICU stays

The coronavirus will not kill most people it infects, yet many of those infected will bear the cost of health-care services, which may be considerable in the aggregate if a significant number are hospitalized, are admitted to an ICU, or, in the most extreme cases, require mechanical ventilation. Many adults will also develop symptoms of COVID-19 that may not require hospitalization but that will require them to miss work. In this section, we estimate the net effect of suppression policies in terms of reducing symptomatic infections, hospitalizations, ICU stays, and mechanical ventilation.

In order to estimate the total expected number of symptomatic infections among U.S. adults under suppression measures, we consider estimates of the IFR and the share of COVID-19 cases that are asymptomatic. First, we estimate the IFR for each age group reported in the CDC’s death data [13] by using a metaregression equation that estimates a log-linear relationship between age and IFR [15]. Dividing the number of deaths observed in each age group by each age group’s estimated IFR allows for estimation of the total number of infections in the U.S. as of the week ending August 1, 2020.

We estimate that 25.8 million U.S. adults were infected with COVID-19 as of that week, which is approximately 10.4 percent of the U.S. adult population [16]. This is comparable to the result of a U.S. seroprevalence study that finds that approximately 9.3 percent of all U.S. adults, or 23.6 million adults, had been infected by COVID-19 as of July 2020 [17]. Our calculations are presented in Table 2. Notably, our results imply an adult population IFR of 0.6 percent, which is within one-tenth of a percent of some previous IFR estimates [18, 19].

**Table 2:** Estimated COVID-19 infections among U.S. adult population, week ending August 1, 2020.

Age	Midpoint of Age Group	Deaths	Predicted IFR	Estimated Infections
18 to 24	21.0	298	>0.0%	4,403,685
25 to 34	29.5	1,317	>0.0%	6,978,881
35 to 44	39.5	3,326	0.1%	5,273,783
45 to 54	49.5	8,622	0.2%	4,090,798
55 to 64	59.5	20,378	0.7%	2,893,087
65 to 74	69.5	34,272	2.4%	1,455,926
75 to 84	79.5	42,143	7.9%	535,705
85 plus	89.5	50,446	26.3%	191,879
**Total**	—	**160,802**	**0.6%**	**25,823,744**

*Sources*: [13, 15]; authors’ calculations.

A significant share of COVID-19 infections are believed to be asymptomatic, though there is substantial uncertainty about what share of COVID-19 cases never show symptoms. Mizumoto et al. estimate that about 18 percent of passengers aboard the *Diamond Princess* cruise ship who had confirmed COVID-19 infections were asymptomatic [20]. Meanwhile, the CDC estimates that 30 percent of people infected by coronavirus in the United States may never develop symptoms [21]. These estimates of the share of infections that are asymptomatic imply that between 70 percent and 82 percent of infections are symptomatic. As such, we estimate that, in total, between 18.1 million and 21.2 million U.S. adults experienced symptomatic COVID-19 infections as of August 1, 2020.

With respect to hospitalizations, through the week ending August 1, 2020, CDC estimates that cumulatively 179.7 adults aged 18 and older were hospitalized due to COVID-19 for every 100,000 adults in the U.S. population [12]. Multiplying 179.7 per 100,000 adults by the total U.S. adult population yields approximately 448,000 total hospitalizations for U.S. adults [16]. According to the CDC, 45,682 adults were hospitalized with coronavirus in the COVID-NET Network between the week ending March 7, 2020, and the week ending August 1, 2020 [12]. Assuming each age group’s share of hospitalizations in the COVID-NET Network is the same as its share of total hospitalizations across the country, we estimate the total number of COVID-19 patients hospitalized in the United States during that time period by age group. Those estimates are shown in Table 3. The CDC also estimates that 18.9 percent of 18 to 49 year-olds, 27.1 percent of 50 to 64 year-olds, and 26.9 percent of people 65 and older of those who are hospitalized for COVID-19 are admitted to the ICU [21]. Similarly, CDC estimates the percentage of hospitalized COVID-19 patients that require mechanical ventilation for the same three adult age groups [21]. Its estimates are: 9.0 percent of hospitalized 18 to 49 year-olds; 15.1 percent of 50 to 64 year-olds; and 15.6 percent of those 65 and older. Table 3 presents our calculation of the total number of adults hospitalized, admitted to ICU, and requiring mechanical ventilation in the U.S., broken down by the CDC’s age groups.

**Table 3:** Estimated COVID-19 hospitalizations for U.S. adults, week ending August 1, 2020.

Age	Number of COVID-19 hospitalizations, COVID-NET	Share of COVID-19 hospitalizations, COVID-NET	Estimated total hospitalized, by age group	Estimated total ICU admissions, by age group	Estimated mechanical ventilation, by age group
18 to 49	13,434	29.4%	131,770	24,905	11,859
50 to 64	13,324	29.2%	130,691	35,417	19,734
65 plus	18,924	41.4%	185,620	49,932	28,957
**Total**	**45,682**	**100.0%**	**448,082**	**110,254**	**60,550**

*Sources*: [12, 16, 21]; authors’ calculations.

*Note*: Sums may not be exact owing to rounding.

The above estimates reflect our best approximation of what occurred under government suppression policies. To estimate the number of symptomatic infections, hospitalizations, ICU admissions, and uses of mechanical ventilation under our counterfactual scenario, we assume the relationships among these factors in the suppression scenario hold in the counterfactual scenario. We infer the number of adults that have symptomatic cases of COVID-19 under the counterfactual scenario by dividing the estimated number of deaths by the infection fatality rate for symptomatic cases (IFR-S) for U.S. adults. We estimate the IFR-S for U.S. adults by dividing the observed number of COVID-19 deaths among adults (160,802) by our estimated range of total symptomatic infections (18.1 to 21.2 million), which yields an IFR-S of between 0.8 percent and 0.9 percent. Ferguson et al. projected 1.1 million to 1.2 million COVID-19 deaths in the United States under its “most effective” mitigation scenario [2]. Dividing 1.1 million deaths by the upper-bound IFR-S estimate (0.9 percent) and 1.2 million by the lower-bound IFR-S estimate (0.8 percent), we estimate that between 122 million and 150 million people in the United States would need to have been infected and developed symptoms of COVID-19 in order for that number of deaths to occur.

To infer the number of hospitalizations, ICU admissions, and mechanically ventilated patients in the mitigation scenario, we observe that approximately 2.5 percent of the 18.1 million adults who had symptomatic cases of COVID-19 are hospitalized, and about 24.6 percent and 13.5 percent of hospitalizations result in ICU admission and mechanical ventilation, respectively (see the figures in Table 3). If 122 million to 150 million adults have symptomatic cases, then between 3.1 million and 3.8 million adults would be expected to be hospitalized in the U.S. Of those hospitalized, between 760,000 and 930,000 would be admitted to the ICU, and between 420,000 and 510,000 COVID-19 patients would require mechanical ventilation.

Table 4 summarizes our health-care utilization estimates under the counterfactual private mitigation scenario and the observed suppression scenario. Calculating the difference between our estimates under these scenarios allows us to estimate the net effect of suppression policies.

**Table 4:** Estimates of the net effects of COVID-19 suppression measures on health-care utilization among U.S. adults, as of the week ending August 1, 2020.

Category	Private mitigation scenario	Government suppression scenario	Net effect of suppression measures
COVID-19 symptomatic infections	122–150 million	18.1–21.2 million	101–132 million
Hospitalizations	3.1–3.8 million	448,000	2.7–3.4 million
ICU admissions	760,000–930,000	110,000	650,000–820,000
Mechanical ventilation	420,000–510,000	61,000	360,000–450,000

*Sources*: [2, 12, 13, 15, 20, 21]; authors’ calculations.

*Note*: Differences or sums may not be exact owing to rounding.

#### Estimated cost of health-care utilization

Many adults who contract COVID-19 will not be hospitalized but will bear the cost of lost wages. The CDC advises those with COVID-19 to isolate for at least 10 days, and to remain isolated until fever and other symptoms improve [22]. We calculate the cost of a case of COVID-19 treated at home to be approximately equal to two weeks of lost earnings, which, on average, is just over $1,900. We calculate this by multiplying the average hourly wage in January 2020, $28.43 [23], by the average number of hours worked by an “engaged person” in two weeks during 2017, which was about 68 hours [24].

For those who develop more serious symptoms or develop complications, hospitalization may be necessary. To approximate the cost of a hospitalization from COVID-19, we use the estimate from Torio and Moore for the average cost of a hospitalization for pneumonia [25]. Like COVID-19, pneumonia is a respiratory condition, and it is common for COVID-19 patients to develop pneumonia in mild and severe cases [26]. Adjusted to 2020 dollars, the average cost of a pneumonia hospitalization was just over $11,000. This estimate likely overestimates the costs of non-ICU hospitalizations because it may include those patients who spend time in the ICU. That said, we will use this number since it is the best estimate available, with the understanding that it might overestimate the average cost of non-ICU hospitalizations, since ICU stays have substantially higher costs than a standard hospitalization, especially on the first day.

We break down other health-care utilization costs according to how many hospitalized patients require an ICU stay and how many require mechanical ventilation. We use estimates from Dasta et al. on average costs for ICU patients and for patients needing mechanical ventilation [27]. We source estimates of median ICU length of stays and median number of days of mechanical ventilation for COVID-19 patients from CDC [21], which presents estimates of the median number of days of hospitalization for patients admitted to the ICU by the same three adult age groups—10 days for those 18 to 49, 14 days for those 50 to 64, and 13 days for those 65 and older. Calculating an average of the three median estimates weighted by the number of observed hospitalized patients in each age group (see Table 3), we estimate that an adult admitted to the ICU will have an expected length of stay of just over 12 days. Dasta et al. estimate, all in 2002 dollars, that the first day in the ICU costs about $6,700, the second day costs about $3,500, and each ICU day thereafter costs about $3,000 [27]. Taking our estimate of a median stay of 12 days for ICU patients, average ICU costs are $40,200 in 2002 dollars, or about $58,500 in 2020 dollars.

For the 13.5 percent of hospitalized COVID-19 patients that we estimate will require mechanical ventilation, they are expected to require 5 days of mechanical ventilation [21]. Dasta et al. estimate that the first day of mechanical ventilation costs about $11,000, the second day about $5,000, and $4,000 thereafter, again in 2002 dollars [27]. We estimate costs for five days of mechanical ventilation during a stay that lasts 12 days. For the seven days without mechanical ventilation, we assume daily costs of $3,000, which is the daily cost of a standard ICU day. Altogether, we estimate average ICU costs of $49,000 (in 2002 dollars), which is about $71,400 in 2020 dollars.

Above, we presented estimates of the effects of suppression measures relative to the baseline scenario of more targeted mitigation practices, in terms of the expected reductions in COVID-19 infections in which symptoms are present, hospitalizations, ICU admissions, and mechanical ventilation. We calculate the financial value of these benefits by simply multiplying the number of people predicted to be relieved of each medical service by the average economic cost of that service. In the case of symptomatic illnesses, we multiply the estimated number of people affected by two weeks of average earnings. We find that the benefits associated with prevented illnesses and reduced health-care utilization are between $285.3 billion and $368.3 billion.

#### Reduced incidence of lung damage

Some patients who have recovered from COVID-19 develop acute respiratory distress syndrome (ARDS) and may have permanent lung damage and decreased lung capacity. Zhou, Yu, et al. find that 9 of 137 (6.6 percent) COVID-19 survivors in Wuhan who were ultimately discharged from the hospital developed ARDS [28].

To estimate the number of people who will be impacted by ARDS as a result of COVID-19, we assume that 6.6 percent of those who are hospitalized and recover will develop ARDS. We simply subtract the number of expected deaths from the range of estimates of expected total hospital admissions from above, and then multiply that number by 6.6 percent. Doing so, we estimate that between 125,000 and 178,000 ARDS cases would emerge under a targeted mitigation strategy, while only about 19,000 ARDS cases are expected under suppression policies. This means suppression measures may reduce ARDS cases resulting from COVID-19 by between 106,000 and 159,000.

A 2017 study of ARDS patients in the United States measured their use of inpatient and outpatient services within the first year of their diagnosis of ARDS. Ruhl et al. find that 55 percent of the ARDS patient cohort in the study sought inpatient services (e.g., hospitalization or skilled nursing facility) at a median cost of $16,800, in 2014 dollars. Meanwhile, 88 percent of the cohort sought outpatient services from a primary care physician or specialists, such as a pulmonologist, at a median cost of $6,761, also in 2014 dollars [29]. In expected-value terms, an ARDS patient will bear approximately $16,700 in inpatient and outpatient costs within the first year, after adjustment to 2020 dollars.

Considering only first-year health-care costs likely understates the expected total health-care costs for those who develop ARDS as a result of COVID-19. Further, permanent lung damage also likely has a significant effect on recovered patients’ productivity for the remainder of their life. As such, we assume those who develop ARDS will see their lifetime total production decrease by 30 percent. Using the same Grosse et al. estimates of total lifetime production by age [14], we calculate the expected lifetime production lost for those hospitalized with COVID-19 who develop ARDS (see S1.2 Table in S1 Appendix). We weight this average by the distribution of age among 45,682 adults hospitalized in the COVID-Net Network [12]. Results are presented in Table 5.

**Table 5:** Expected lifetime production lost to lung damage resulting from COVID-19.

Age	Lifetime production, 2020 USD	Number of COVID-19 hospitalizations, COVID-NET	Approx. share of COVID-19 hospitalizations	Expected lifetime production lost (30% reduction)
18 to 49	$1,571,597	13,434	29.4%	$138,651
50 to 64	$746,446	13,324	29.2%	$65,314
65 plus	$234,035	18,924	41.4%	$29,085
**Total**	**—**	**45,682**	**100.0%**	**$233,050**

*Sources*: [12, 14]; authors’ calculations.

We estimate that a patient who recovers from COVID-19 but develops ARDS will, on average, see a loss of just over $233,000 to his or her lifetime production. Combined with the expected cost of care in the first 12 months ($16,700), we estimate the present value of total costs of lung damage to be just under $250,000. Multiplying that cost estimate by the 106,000 to 159,000 people who we expect won’t develop ARDS as a result of suppression measures, we estimate the economic benefit of reduced permanent lung damage from suppression measures to be between $26.5 billion and $39.8 billion.

#### Aggregate gross benefits of COVID-19 suppression measures

To summarize, we expect that the primary benefits of policies that slow the spread of the novel coronavirus will be reduced mortality, reduced symptomatic infections leading to lost earnings, reduced health-care utilization in the form of hospitalizations, ICU stays, and mechanical ventilation, and reduced permanent lung damage among a subset of those who contract and recover from COVID-19. Compared with the outcomes projected under Ferguson et al.’s model of the most effective mitigation practices (the no-suppression policy counterfactual) [2], including case isolation, household quarantine, and social distancing among elderly individuals and high-risk populations, we estimate total gross benefits in the range of $629.5 billion to $759.6 billion. Note that the mortality reduction benefits associated with suppression measures are gross estimates and do not yet account for any increases or decreases in mortality risk that might accompany economic dislocations or cost savings. We return to this issue shortly.

### Cost analysis

#### Forgone output

A shock to economic output would be expected regardless of what policies the government enacts in response to the outbreak of COVID-19. Relative to a baseline of continued pre-pandemic economic activity, Mulligan estimates that the impacts of shutting down nonessential activities during the pandemic have total welfare costs of $1,768 billion on a quarterly basis [30]. An even more pessimistic forecast from Makridis and Hartley estimates total losses in GDP of just over $2 trillion during the first two months of the COVID-19 outbreak in the United States (April and May 2020) [31]. However, both estimates are of the total economic costs of private and public measures to slow the spread of COVID-19. Moreover, real GDP only decreased about 3.5 percent, or a little under $800 billion, in 2020 relative to the 2019 level [32]. Even assuming fairly rapid growth would have occurred in absence of the pandemic, these estimates likely overestimate losses to economic production during the early pandemic period.

The key challenge in calculating the costs of suppression measures is isolating the costs of policy from the costs of private action undertaken to mitigate risks during the pandemic. Scherbina [33] estimates that the incremental cost of suppression policies, relative to Ferguson et al.’s [2] mitigation scenario, is approximately $35.8 billion per week, or about $5.1 billion per day, on average. According to this estimate, suppression policies may have imposed economic costs of $143 billion every four weeks and $465 billion every quarter. This implies that enforcing suppression policies for an entire year (as opposed to the much shorter period that they were actually in force) would have resulted in GDP losses of approximately 8.7 percent of 2019 GDP.

To estimate the aggregate costs of state-level suppression polices, we calculate the number of days during which the U.S. states enforced stay-at-home orders and nonessential business closures. Requiring residents to stay at home and requiring nonessential businesses to close are not the only suppression policies, but they likely imposed the most costs on economic output among the NPIs that were widely enforced during the initial outbreak of COVID-19. The start and end dates of these orders for each state are sourced from IHME [1] and listed in S2.1 Table in S2 Appendix.

We weight the number of days that each state enforced stay-at-home orders and nonessential business closures by each state’s GDP relative to U.S. GDP as of the fourth quarter of 2019 [34]. Weighting the number of suppression days by GDP reflects the fact that a day of suppression in a larger state, such as California, causes more lost output than a day of suppression in a smaller state, such as Maine. We then sum across states to calculate a weighted average of the number of days nonessential business closures and stay-at-home orders were in place.

Distinguishing the incremental costs specific to a stay-at-home order from the incremental costs specific to a nonessential business closure order (as well as distinguishing the incremental costs of when these policies are enforced jointly) is a difficult task. Accordingly, we calculate two weighted averages to produce lower- and upper-bound estimates of the number of days in which suppression policies were enforced in the United States.

First, we calculate the number of days that both a stay-at-home order and a nonessential business closure order were enforced at the same time. For the 22 states that did not enforce both types of legal orders jointly, we set the number of days of suppression equal to zero. In this lower-bound scenario, we estimate that U.S. states enforced suppression, on average, for 42 days, equal to 6 weeks. That calculation is shown in S2.2 Table in S2 Appendix.

Second, we calculate the number of days that *either* a stay-at-home order or a nonessential business closure order was enforced by states. For the six states that did not enforce either measure, we set the number of days of suppression policies equal to zero. In this upper-bound case, we estimate that suppression policies were enforced for an average of 65 days, which is just over 9 weeks. That calculation is shown in S2.3 Table in S2 Appendix.

Multiplying the range of the estimated number of days in which the U.S. states enforced suppression policies (42–65 days) by the estimated daily incremental cost of suppression policies ($5.1 billion), we estimate that these state policies resulted in losses to economic output between $214.2 billion and $331.5 billion.

#### Countervailing risks from lost income

As a result of the COVID-19 pandemic, the United States faced the prospect of a prolonged economic downturn. Economic dislocation can impose costs not just on household finances, but also on health and safety. While the effects of the business cycle on mortality can be positive or negative, depending on the risk being considered, the effects of lost income over the long term tend to have unambiguous detrimental effects on health. When incomes fall enough, deaths can be expected. The mechanism driving this effect is that economic costs reduce expenditures made by households to reduce risk privately. A recent estimate suggests that for every $111 million (in 2020 dollars) in reduced income, one expected death will occur [10].

However, ancillary changes in mortality risks owing to income shocks can be positive or negative, depending on whether policies on balance impose costs (thereby reducing incomes) or reduce costs (thereby increasing incomes). The total cost estimate of suppression policies above ranges from about $214.2 billion to $331.5 billion. Costs of $214.2 billion to $331.5 billion would correspond to an initial 1,900 to 3,000 additional expected deaths. However, total gross benefits are estimated to be in the range of $629.5 billion to $759.6 billion. Because these benefits come in the form of cost-savings or prevention of lost production (and by extension prevention of lost income), they result in offsetting coincident risk *reductions*, estimated at 5,700 to 6,800 initial lives saved. The net effect, therefore, of these ancillary risks, has a present value that ranges from 2,700 to 4,900 additional expected lives saved.

We estimate a production value of the average American of approximately $1.1 million (see S1.3 Table in S1 Appendix). Assuming these changes in ancillary risks are spread equally across the entire U.S. population, the gains to production associated with 2,700 additional prevented deaths would be approximately $3.0 billion in present value terms, and the present value of the benefit associated with 4,900 fewer expected deaths is $5.4 billion.

Taken together, the ancillary mortality risks associated with income changes may actually produce additional benefits valued between $3.0 billion and $5.4 billion. Combining these estimates with the gross mortality benefits estimated in the section on reduced mortality ($317.7 billion to $351.5 billion), we estimate net mortality benefits between $320.7 billion and $356.9 billion. The benefits associated with extending lives through this income-mortality channel should not be confused with benefits from preventing COVID-19 deaths, which are calculated separately above. Rather, these benefits stem from preventing deaths that would have followed from the financial ravages of the pandemic, which are likely to play out over a different time horizon and affect a separate, and probably younger on average, class of individuals than those who die of COVID-19.

### Net benefits

We estimate the net benefits of COVID-19 suppression policies in the United States enforced between March and August 2020 are between $301 billion and $550.8 billion. Table 6 shows our estimates of net effects, per person prevented costs, and aggregate benefits, as well as our estimate of forgone GDP due to stay-at-home orders and nonessential business closures. The most significant factor in our estimate of benefits is reduced mortality. In fact, the lower bound of our estimate of the net mortality benefits almost surpasses the upper-bound estimate of total costs. Considering the benefits of reduced mortality alone, suppression policies as they were enforced during the summer months of 2020 in the United States likely pass a cost-benefit test. That said, the other factors for which we estimate benefits of suppression policies also produce significant benefits.

**Table 6:** Net benefit estimates of COVID-19 suppression measures enforced from early March 2020 to August 1, 2020.

Category	Effect relative to baseline	Value per person, 2020 USD	Value, 2020 USD
Net reductions in mortality	940,000–1.04 million	—	$320.7–$356.9 billion
Prevented COVID-19 deaths	940,000–1.04 million	$338,000	$317.7–$351.5 billion
Initial deaths from lost income	(4,900)–(2,700)	$1.1 million	($5.4)–($3.0 billion)
COVID-19 symptomatic infections	101–132 million	$1,900	$191.9–$250.8 billion
Hospitalizations	2.7–3.4 million	$11,000	$29.7–$37.4 billion
ICU admissions	650,000–820,000	$58,500	$38–$48 billion
Mechanical ventilation	360,000–450,000	$71,400	$25.7–$32.1 billion
ARDS cases	106,000–159,000	$250,000	$26.5–$39.8 billion
**Total benefits**	—	—	**$632.5–$765.0 billion**
**Total costs**	—	—	**$214.2–$331.5 billion**
**Net benefits**	—	—	**$301.0–$550.8 billion**

*Source*: Authors’ calculations.

*Note*: Sums may not be exact, owing to rounding.

### Alternative mortality benefit estimations

#### The value of a statistical life (VSL)

Many studies estimating the value of the reduced mortality utilize the VSL. In order to use the VSL, the analyst must first decide which individual’s or group’s preferences should dictate policy choices, and that choice can dramatically change the VSL [35]. Viscusi recommends an average VSL of $11 million (in 2019 dollars), or $11.3 million when adjusted to 2020 dollars [36]. This VSL is based primarily on the preferences of current workers. Meanwhile, the VSL might also be adjusted downward for the relatively older population affected by COVID-19, or for the fact that the level of risk most Americans face from COVID-19 is larger than that found in many of the studies that estimate the VSL [37]. However, one could go further and choose to adjust the calculation to account for altruistic motives of family members or friends [38]. Going even further, children, the unborn, and future generations, are also affected by policy but their preferences are not reflected in present-day markets. Any changes in assumptions to account for these groups’ preferences, and by extension their willingness to pay, can change the corresponding VSL estimate dramatically.

Given a general lack of consensus in this area, we estimate the mortality benefits of prevented COVID-19 deaths using a U.S. population average VSL of $11.3 million [36]. Our estimates above suggested that suppression policies prevented 940,000 to 1.04 million deaths in the U.S. between March 2020 and August 1, 2020, and that an additional 2,700 to 4,900 initial lives would be saved due to cost-savings. Thus, net mortality benefits are recalculated to be between $10.6 trillion and $11.9 trillion, or roughly half of 2019 U.S. GDP when based on the VSL.

Reestimating mortality benefits in this way clearly overwhelms estimates of other benefits and costs in our analysis. However, inferring from these estimates that suppression policies therefore had trillions of dollars in net benefits is inappropriate. Our cost estimates would need to be recalculated as well to include non-pecuniary factors, such as reduced time spent with family and friends. In addition, other benefits associated with prevented health-care utilization might need to be adjusted to be based on the current population’s willingness to pay to avoid those services and illnesses.

#### Quality-adjusted life years (QALYs)

Prevention of lost life years is another way to evaluate the effectiveness of suppression policies. To estimate expected life years saved, we use the United States Life Tables for 2018 [39]. In expected value terms, people who died of COVID-19 up to August 1, 2020 lost 13.8 life years (see S1.4 Table in S1 Appendix). In an analysis that adjusts life years for quality of life factors, such as comorbidities, Briggs et al. estimate that the average quality-adjusted life expectancy of a person who died of COVID-19 in the U.S. as of July 2020 would have been between 6.1 and 10.2 years [40]. This estimate of quality-adjusted life expectancy is equivalent to undiscounted QALYs. Using our previous estimate of between 940,000 and 1.04 million prevented COVID-19 deaths, we calculate a total mortality benefit of between 5.7 million and 10.6 million QALYs saved. Without quality adjustments, total life years saved are approximately 14.4 million.

A common monetary threshold for a QALY employed in cost-effectiveness studies is $50,000 [41]. This value per QALY implies gross mortality benefits of between $285 billion and $530 billion. Interestingly, our preferred estimate of gross mortality benefits using the “value-of-production” approach falls entirely inside this interval, suggesting similarities between the QALY and the value-of-production approaches.

Like with the calculations using the VSL, a limitation of this QALY analysis is that we do not consider the benefits associated with prevented illness or lung damage in the same units, in this case in terms of QALYs. Nevertheless, we believe presenting these estimates are informative for comparison purposes and for accounting for uncertainty surrounding the value of life that is appropriate in the context of the COVID-19 pandemic, as well as in general.

## Discussion

Our finding of positive net benefits is consistent with other CBAs of social distancing during the pandemic [3, 4], though our net benefit estimates are smaller in magnitude. The distinction in our findings is attributable to two key contributions of our article to this literature. First, we attempt to estimate the costs and benefits associated with the *policy response* to COVID-19, not the costs and benefits associated with social distancing more generally, which includes public and private actions that reduce the health and economic impacts of COVID-19. Second, our analysis focuses on the costs and benefits of COVID-19 suppression in terms of its effects on economic output and production.

Our value-of-production approach to valuing mortality benefits allows for more direct comparisons of mortality benefits to other relevant benefits and costs. For example, health-care utilization is most easily measured in terms of its observed cost of service. Similarly, costs associated with policy interventions, such as losses to GDP, are comparable to these production benefits. Moreover, our focus on production is similar to other CBAs in the literature, such as one evaluating COVID-19 screening tests [42].

Another novel contribution of our CBA is that it explicitly accounts for potential increases in mortality risks, owing to the economic costs associated with income losses stemming from public policies [10]. Because we find that suppression policies are, on net, cost-saving, we find that these policies prevented additional deaths through this income-saving channel, in addition to preventing COVID-19 deaths in a direct manner. While the indirect mortality benefits are small relative to benefits overall, we believe there is value in calculating these ancillary mortality risks due to concerns that the effect of suppression policies on factors such as loneliness and depression could be significant in magnitude and possibly even outweigh the health consequences of COVID-19 itself. By contrast, our findings suggest that suppression policies had minimal short-run effects on overall mortality through these indirect channels. Although economic dislocation is not the only factor that may have affected mental health while suppression policies were enforced, our results are consistent with a recent study that found the number of suicides in several US states and high-income countries during the summer and fall months of 2020 did not deviate significantly from pre-COVID-19 trends [43].

Our analysis has certain limitations as well. One of the most significant is uncertainty regarding the number of COVID-19 deaths that would have occurred in the counterfactual scenario in which suppression policies were not enforced. Our choice of counterfactual is the forecast of COVID-19’s progression in the United States by Ferguson et al. [2], which was published in March 2020 and based on early evidence about disease transmission and mortality. Choosing this counterfactual scenario has some advantages. The time period during which “a single, relatively short epidemic” occurs in the Ferguson et al. [2] forecast closely corresponds to the time period during which almost all U.S. states enforced NPIs—that is, early March 2020 to early August 2020. Further, Scherbina [33] estimates the weekly incremental costs of suppression policies also using Ferguson et al.’s [2] definitions of mitigation and suppression, which allows us to compare our benefit and cost estimates to the same baseline for a consistent measure of net benefits. Finally, Ferguson et al. forewarned that relaxing suppression policies prior to the population reaching herd immunity by vaccination would likely result in increases in illnesses and deaths because a significant share of the population would be vulnerable to infection and illness [2]. Indeed, many U.S. states eased NPIs after August 2020, prior to the distribution of a coronavirus vaccine, and COVID-19 deaths among U.S. adults rose from 160,802 as of August 1, 2020 to over 500,000 as of the week ending February 13, 2021 [13]. While COVID-19 deaths did not increase to over 1 million in the U.S. after NPIs were eased in August 2020, important changes could explain why the winter and fall waves were less severe than the summer wave predicted by Ferguson et al. [2]. First, testing capacity was much more robust in the fall and winter months, which allowed cities and states to more closely monitor disease spread and make targeted interventions when cases increased [44]. Second, physicians learned better techniques for treating COVID-19 patients, and this likely contributed to reduced mortality rates [45, 46].

However, our counterfactual scenario also has limitations. In particular, behavioral responses that affect transmission in the Ferguson et al. model are limited to case isolation, voluntary quarantine for households, and social distancing for at-risk groups [2]. Importantly, the CDC did not recommend the general public wear masks until April 2020 [47], after the publication of Ferguson et al. [2]. Mask wearing has been shown to be an effective NPI for slowing community spread of COVID-19 [48], and adherence to policies or health guidance to wear masks has been shown to correlate with reduced COVID-19 case rates [49]. In addition, compliance with quarantine and social distancing is also treated as a constant. By contrast, behavioral SIR and behavioral SEIR epidemiological models may offer more accurate forecasts of transmission and mortality by accounting for possible endogeneity between transmission rates and the daily death rate [50]. For example, Atkeson finds that a behavioral SEIR model that accounts for seasonal variation in transmission and “pandemic fatigue” (a reduction in the elasticity of social distancing behavior to changes in daily death rates) closely fits observed daily COVID-19 death rates in the U.S. for 2020 [51].

In general, the behavioral response to the daily death rate or some other measure of disease prevalence or severity creates uncertainty about both our cost and benefits estimates. We attribute most social distancing behaviors that reduce infections, health-care utilization, and deaths to public policy interventions. This might be reasonable if the costs of infection are not fully internalized by U.S. adults. However, studies of geographic mobility data have found that major changes in mobility preceded the implementation of stay-at-home policies [52], and another analysis finds that increases in mobility are better explained by reductions in daily death rates than the easing of stay-at-home orders [53]. In other words, in a counterfactual scenario in which policies were eased despite daily deaths remaining high, social interaction and mobility may have remained low. If the private response to COVID-19 more closely resembles population-wide social distancing than targeted private mitigation as described in Ferguson et al. [2], then the incremental costs and benefits of suppression policies reported in this article are likely both overstated.

Our computational methods also have limitations and could introduce bias to our estimates. For one, we assume that the population IFR is equal in the mitigation and suppression scenarios. Our use of the IFR implied by the number of deaths and infections under suppression policy enforcement may understate the age-specific and population IFR that would occur under mitigation given capacity constraints in the health-care system, such as the limited number of ICU beds and mechanical ventilators. Underestimating the IFR in the counterfactual scenario would imply that our estimated number of symptomatic infections among U.S. adults in the counterfactual scenario are overestimated, which would bias our benefits estimates upward.

We also don’t consider supply constraints in our estimates of health-care utilization and assume equal proportional relationships in hospitalizations, ICU stays, and mechanical ventilation between the suppression and mitigation scenarios. As described by Ferguson et al., the forecasted mitigation scenario would result in at least some regions of the U.S. facing shortages in both available ICU beds and mechanical ventilators for COVID-19 patients [2]. Our analysis, meanwhile, does not estimate when demand for hospital and ICU beds or mechanical ventilators would exceed their supply in the U.S., or how many COVID-19 patients would not have access to health-care services as a result. In light of this limitation, we assume that all COVID-19 patients who need to be hospitalized, receive intensive care treatment, or require mechanical ventilation obtain those services. Accordingly, our estimates of hospital admissions, ICU stays, and mechanical ventilation could be overstated in the mitigation scenario if some individuals who would need these services would not have access to them. The overall death count would remain unchanged because Ferguson et al. [2] account for the possibility of medical supply shortages, but this assumption could bias our other estimates of benefits upwards (and by extension our estimate of net benefits upwards) because medical utilization could be less in the counterfactual scenario due to binding constraints in available health-care resources.

Our bottom-up approach of aggregating factors that have incremental effects on production omits certain factors that could also affect production. For instance, we consider the potential long-term effects of lung damage among recovered COVID-19 patients, but other potential chronic conditions have been observed following recovery from COVID-19 infection, such as myocarditis [54] and neurological effects [55, 56]. Estimating the prevalence of these conditions and their long-term effects is difficult only a short while after the coronavirus began spreading in the U.S. Moreover, NPIs have resulted in certain unintended consequences. Studies have identified upticks in domestic violence as a potential result of sheltering at home [57], as well as reductions in traffic accidents [58].

While we do not expect those factors would dramatically change our conclusions, even the factors accounted for in our study may not be measured precisely. For instance, we assume a permanent loss in remaining lifetime total production, but a cohort study of Severe Acute Respiratory Disorder (SARS) patients found that one year after hospital discharge, over half showed no signs of lung impairment [59]. Declines in some forms of market production, such as child care or restaurant dining, could be made up for by nonmarket production in the household, such as homeschooling, making dinner at home, or caring for a sick family member. In this sense, estimates of changes in output could overestimate the costs of suppression policies. On the other hand, declines in research and development expenditures or investments in human capital may impose total costs that exceed short-term losses to output. For instance, closing schools for only a few months could result in reduced earnings over the lifetimes of affected children [60].

Finally, as noted above, there is uncertainty surrounding what valuation method to employ once the quantity of various benefits and costs has been calculated. Uncertainty about the value of life is the most obvious example. However, there is also uncertainty more generally about whether market prices for health-care services accurately correspond with the value of resources in terms of their opportunity cost to society. In spite of these challenges, we have done our best to include what we believe are the most direct, impactful, and predictable effects of suppression policies.

## Conclusion

We estimate that suppression policies enforced by the U.S. states in the spring and early summer months of 2020 had substantial net benefits, in terms of preventing losses to economic output. Relative to targeted mitigation strategies that would likely have been adopted instead of suppression policies, we estimate that the benefits of suppression policies that bent the curve of COVID-19 are between $632 billion and $765 billion through August 1, 2020. However, we find that suppression policies resulted in substantial losses to GDP, too, between $214 billion and $332 billion. Our results suggest that the net benefits of suppression policies on total economic production are positive and likely substantial, possibly as high as $551 billion.

These estimates assume that the bending of the curve of COVID-19 cases and deaths in the United States is largely attributable to suppression policies that were implemented in most U.S. states. However, if the American public would have engaged in social distancing irrespective of state-enforced NPIs, then the benefits and costs of these policies may be much lower. In that case, our estimates can be thought of as estimates of the costs and benefits of social distancing broadly, which includes private actions. To gain a better understanding of the effectiveness of NPIs to address COVID-19, further research that examines the relationship between specific COVID-19 policy interventions and disease transmission would be beneficial.

## Supporting information

S1 Dataset Spreadsheet with benefits and costs calculations. (XLSX)

S1 Appendix Value-of-life tables. (DOCX)

S2 Appendix U.S. state suppression policies to slow the first wave of COVID-19. (DOCX)
