# Supplementary material for: The benefits of coronavirus suppression: A cost-benefit analysis of the response to the first wave of COVID-19 in the United States
Source: PLoS One. 2021 Jun 3;16(6):e0252729. doi: 10.1371/journal.pone.0252729 (PMC8174714; doi:10.1371/journal.pone.0252729)
Supplement: S1 Appendix — (DOCX) [file pone.0252729.s002.docx]

**S1 Appendix. Value-of-life tables.**

**Table S1.1. Author calculations of the present value of lifetime production by age, adjusted to age groups in CDC COVID-19 deaths counts.**

| **Age** | **Lifetime total production, 2007 USD** | **Lifetime total production, 2020 USD** | **CDC COVID-19 deaths age groups** | **Lifetime total production, 2020 USD** |
| --- | --- | --- | --- | --- |
| 20 to 24 | $1,119,137 | $1,700,684 | 18 to 24 | $1,700,684 |
| 25 to 29 | $1,164,022 | $1,768,893 | 25 to 34 | $1,743,368 |
| 30 to 34 | $1,130,428 | $1,717,842 |  |  |
| 35 to 39 | $1,051,137 | $1,597,348 | 35 to 44 | $1,511,338 |
| 40 to 44 | $937,939 | $1,425,328 |  |  |
| 45 to 49 | $802,484 | $1,219,486 | 45 to 54 | $1,102,485 |
| 50 to 54 | $648,498 | $985,483 |  |  |
| 55 to 59 | $486,469 | $739,257 | 55 to 64 | $626,928 |
| 60 to 64 | $338,632 | $514,598 |  |  |
| 65 to 69 | $230,954 | $350,967 | 65 to 74 | $305,058 |
| 70 to 74 | $170,533 | $259,149 |  |  |
| 75 to 79 | $123,803 | $188,136 | 75 to 84 | $163,013 |
| 80 plus | $90,738 | $137,889 |  |  |
|  |  |  | 85 plus | $137,889 |

*Sources*: [13, 14]; authors’ calculations.

*Note*: We use estimates in Grosse et al. [14] that apply a 5 percent discount rate, and then we adjust for inflation using the CPI from January 2007 to January 2020. We also adjust for average annual labor productivity growth, measured in terms of real output per hour, from 2007 to the end of 2019, which was approximately 1.39 percent per year on an annualized basis. Lifetime total production values represent an average of the production values for the age groups from Grosse et al. [14] that the CDC [13] age groups span.

**Table S1.2. Author calculations of the present value of lifetime production by age, adjusted to age groups in CDC hospitalized patient counts.**

| **Age** | **Lifetime total production, 2007 USD** | **Lifetime total production, 2020 USD** | **CDC hospitalization age groups** | **Lifetime total production, 2020 USD** |
| --- | --- | --- | --- | --- |
| 20 to 24 | $1,119,137 | $1,700,684 | 18 to 49 | $1,571,597 |
| 25 to 29 | $1,164,022 | $1,768,893 |  |  |
| 30 to 34 | $1,130,428 | $1,717,842 |  |  |
| 35 to 39 | $1,051,137 | $1,597,348 |  |  |
| 40 to 44 | $937,939 | $1,425,328 |  |  |
| 45 to 49 | $802,484 | $1,219,486 |  |  |
| 50 to 54 | $648,498 | $985,483 | 50 to 64 | $746,446 |
| 55 to 59 | $486,469 | $739,257 |  |  |
| 60 to 64 | $338,632 | $514,598 |  |  |
| 65 to 69 | $230,954 | $350,967 | 65 plus | $234,035 |
| 70 to 74 | $170,533 | $259,149 |  |  |
| 75 to 79 | $123,803 | $188,136 |  |  |
| 80 plus | $90,738 | $137,889 |  |  |

*Sources*: [12, 14]; authors’ calculations.

*Note*: We use estimates in Grosse et al. [14] that apply a 5 percent discount rate, and then we adjust for inflation using the CPI from January 2007 to January 2020. We also adjust for average annual labor productivity growth, measured in terms of real output per hour, from 2007 to the end of 2019, which was approximately 1.39 percent per year on an annualized basis. Lifetime total production values represent an average of the production values for the age groups from Grosse et al. [14] that the CDC [12] age groups span.

**Table S1.3. Expected lifetime production lost to increased mortality from lost national income.**

| **Age** | **Lifetime total production, 2020 USD** | **U.S. population (2018)** | **Approx. percentage of U.S. population (2018)** | **Expected lifetime production, 2020 USD** |
| --- | --- | --- | --- | --- |
| 0 to 4 | $864,396 | 19,836,850 | 6.1% | $53,102 |
| 5 to 9 | $1,052,093 | 20,311,494 | 6.3% | $66,180 |
| 10 to 14 | $1,278,457 | 20,817,419 | 6.4% | $82,422 |
| 15 to 19 | $1,518,608 | 21,204,226 | 6.6% | $99,723 |
| 20 to 24 | $1,700,684 | 22,286,970 | 6.9% | $117,382 |
| 25 to 29 | $1,768,893 | 22,779,537 | 7.1% | $124,788 |
| 30 to 34 | $1,717,842 | 21,788,439 | 6.7% | $115,914 |
| 35 to 39 | $1,597,348 | 20,730,622 | 6.4% | $102,551 |
| 40 to 44 | $1,425,328 | 20,032,588 | 6.2% | $88,426 |
| 45 to 49 | $1,219,486 | 20,827,879 | 6.5% | $78,659 |
| 50 to 54 | $985,483 | 21,761,694 | 6.7% | $66,416 |
| 55 to 59 | $739,257 | 21,611,374 | 6.7% | $49,477 |
| 60 to 64 | $514,598 | 19,675,357 | 6.1% | $31,356 |
| 65 to 69 | $350,967 | 16,409,942 | 5.1% | $17,836 |
| 70 to 74 | $259,149 | 12,125,477 | 3.8% | $9,731 |
| 75 to 79 | $188,136 | 8,549,216 | 2.6% | $4,981 |
| 80 plus | $137,889 | 12,153,946 | 3.8% | $5,190 |
| **Total** | **—** | **322,903,030** | **100.0%** | **$1,114,134** |

*Sources*: [14, 16]; authors’ calculations.

*Note*: Differences or sums may not be exact owing to rounding. Grosse et al. [14] present lifetime production by five-year increments between ages 0 and 80+. The U.S. Census Bureau [16] reports population by age in the same five-year increments, except that it separates those who are 80–84 years old from those 85 or older, so we take the sum of those two groups to align them with the Grosse et al. [14] production estimates. Refer to Table S1.1 and Table S1.2 for adjustments of Grosse et al. [14] for inflation and productivity growth.

**Table S1.4. Expected life years lost to COVID-19 deaths.**

| **Age** | **Midpoint of Age Group** | **Expected Remaining Life Years at Midpoint Age** | **Number of COVID-19 deaths** | **Approx. share of COVID-19 deaths** | **Expected life years lost** |
| --- | --- | --- | --- | --- | --- |
| 18 to 24 | 21.0 | 58.5 | 298 | 0.2% | 0.1 |
| 25 to 34 | 29.5 | 51.0 | 1,317 | 0.8% | 0.4 |
| 35 to 44 | 39.5 | 41.7 | 3,326 | 2.1% | 0.9 |
| 45 to 54 | 49.5 | 32.6 | 8,622 | 5.4% | 1.7 |
| 55 to 64 | 59.5 | 24.1 | 20,378 | 12.7% | 3.1 |
| 65 to 74 | 69.5 | 16.5 | 34,272 | 21.3% | 3.5 |
| 75 to 84 | 79.5 | 9.8 | 42,143 | 26.2% | 2.6 |
| 85 plus | 89.5 | 4.9 | 50,446 | 31.4% | 1.5 |
| **All** | **—** | **—** | **160,802** | **100.0%** | **13.8** |

*Sources*: [13, 39]; authors’ calculations.
