# Supplementary material for: The benefits of coronavirus suppression: A cost-benefit analysis of the response to the first wave of COVID-19 in the United States
Source: PLoS One. 2021 Jun 3;16(6):e0252729. doi: 10.1371/journal.pone.0252729 (PMC8174714; doi:10.1371/journal.pone.0252729)
Supplement: S2 Appendix — (DOCX) [file pone.0252729.s003.docx]

# **S2 Appendix. U.S. state suppression policies to slow the first wave of COVID-19**

The COVID-19 forecast produced by the IHME considers several state-level policies in its model [1]. While the details of each policy vary among the U.S. states, the IHME broadly groups public health interventions into five categories:

- *Stay-at-Home Orders:* 38 states and the District of Columbia enacted a stay-at-home order, 36 of which were lifted between the last week of April 2020 and the first week of July 2020. As of August 1, 2020, only two orders remained in force, in California and Georgia.
- *Public School and University Closures:* All 50 states and the District of Columbia ordered educational facilities to be closed by April 4, 2020, and all these measures remain in force through August 1, 2020.
- *Any Restriction on Size of Gatherings:* 49 states and the District of Columbia placed some legal restriction on public or private gatherings, with the exception being North Dakota. Fifteen states had lifted their restrictions on gatherings entirely on or before August 1, 2020. In total, 34 states and the District of Columbia enforce some restriction on gathering size as of August 1, 2020.
- *Legally Ordered Closure of Any Business:* 49 states and the District of Columbia required at least one type of business (like bars, restaurants, or hair salons) to close starting in late March or early April 2020. Only South Dakota did not actively legally enforce the closure of some businesses in their state. Fifteen states eased business restrictions on or before August 1, 2020, meaning 34 states and the District of Columbia were still enforcing business restrictions through August 1, 2020.
- *Legally Ordered Closure of All Nonessential Businesses:* More restrictive than the category above, 34 states and the District of Columbia ordered all businesses not deemed “essential” to be shut down starting in March or April 2020. By July 3, 2020, all jurisdictions had lifted their nonessential business closures except California, which enforced nonessential business closures as of August 1, 2020.
- *Severe Travel Restrictions:* Only Alaska issued a legal order significantly restricting the travel of its residents within the state, which took effect on March 28, 2020 and remained in effect through August 1, 2020.

The start and end dates of legal orders in each category (except “severe travel restrictions”) are listed by state in Table S2.1. If an order had not been lifted or if an end date had not been formally announced prior as of IHME’s October 29, 2020 update, then the end date is set equal to “to be determined” (TBD), and we assume the policy was in force as of August 1, 2020.

**Table S2.1. Start and end dates of most common policies to enforce social distancing, by state**

|  | **Stay-at-home order** | | **School closures** | | **Gathering size limits** | | **Any business closure** | | **Nonessential business closures** | |
| --- | --- | --- | --- | --- | --- | --- | --- | --- | --- | --- |
| **State** | **Start** | **End** | **Start** | **End** | **Start** | **End** | **Start** | **End** | **Start** | **End** |
| AL | 4/4 | 4/30 | 3/19 | TBD | 3/19 | TBD | 3/19 | 6/15 | 3/28 | 4/30 |
| AK | 3/28 | 4/24 | 3/16 | TBD | 3/24 | 5/22 | 3/17 | 5/22 | 3/28 | 4/24 |
| AZ | 3/30 | 5/16 | 3/16 | TBD | 3/30 | 5/16 | 3/30 | 5/16 | — | — |
| AR | — | — | 3/17 | 8/24 | 3/27 | 6/18 | 3/19 | TBD | — | — |
| CA | 3/19 | TBD | 3/19 | TBD | 3/11 | TBD | 3/19 | TBD | 3/19 | TBD |
| CO | 3/26 | 5/9 | 3/23 | TBD | 3/19 | TBD | 3/17 | TBD | 3/26 | 5/9 |
| CT | — | — | 3/17 | TBD | 3/12 | TBD | 3/16 | TBD | 3/23 | 5/20 |
| DE | 3/24 | 6/1 | 3/16 | TBD | 3/16 | TBD | 3/16 | TBD | 3/24 | 5/8 |
| DC | 3/30 | 5/29 | 3/16 | TBD | 3/13 | TBD | 3/16 | TBD | 3/25 | 5/29 |
| FL | 4/3 | 5/18 | 3/17 | TBD | 4/3 | 6/5 | 3/17 | 9/25 | — | — |
| GA | 4/3 | TBD | 3/18 | TBD | 3/24 | TBD | 3/24 | TBD | — | — |
| HI | 3/25 | 6/10 | 3/19 | TBD | 3/17 | TBD | 3/17 | TBD | 3/25 | 5/1 |
| ID | 3/25 | 5/1 | 3/23 | TBD | 3/25 | 5/1 | 3/25 | 6/13 | 3/25 | 5/1 |
| IL | 3/21 | 5/29 | 3/17 | TBD | 3/13 | TBD | 3/16 | TBD | 3/21 | 5/1 |
| IN | 3/25 | 5/18 | 3/19 | TBD | 3/12 | TBD | 3/16 | 9/26 | 3/24 | 5/18 |
| IA | — | — | 4/4 | TBD | 3/17 | 6/12 | 3/17 | 10/16 | 3/17 | 5/8 |
| KS | 3/30 | 5/4 | 3/17 | TBD | 3/17 | 5/22 | 3/30 | 6/8 | — | — |
| KY | — | — | 3/20 | TBD | 3/19 | TBD | 3/16 | 6/29 | 3/26 | 5/11 |
| LA | 3/23 | 5/15 | 3/16 | TBD | 3/13 | 5/15 | 3/17 | TBD | 3/22 | 5/1 |
| ME | 4/2 | 5/31 | 3/16 | TBD | 3/18 | TBD | 3/18 | TBD | 3/25 | 5/1 |
| MD | 3/30 | 5/15 | 3/16 | TBD | 3/16 | 6/10 | 3/16 | TBD | 3/23 | 5/15 |
| MA | — | — | 3/17 | TBD | 3/13 | TBD | 3/17 | TBD | 3/24 | 5/18 |
| MI | 3/24 | 6/1 | 3/16 | TBD | 3/13 | TBD | 3/16 | TBD | 3/23 | 5/7 |
| MN | 3/28 | 5/18 | 3/18 | TBD | 3/28 | TBD | 3/17 | 6/10 | — | — |
| MS | 4/3 | 4/27 | 3/19 | TBD | 3/24 | TBD | 3/24 | 6/1 | 4/3 | 4/27 |
| MO | 4/6 | 5/15 | 3/23 | TBD | 3/23 | 5/4 | 3/23 | 6/16 | — | — |
| MT | 3/26 | 4/26 | 3/15 | TBD | 3/24 | 6/1 | 3/20 | 6/1 | 3/26 | 5/1 |
| NE | — | — | 4/2 | TBD | 3/16 | TBD | 3/19 | 7/6 | — | — |
| NV | 3/31 | 5/9 | 3/16 | TBD | 3/24 | TBD | 3/18 | TBD | 3/21 | 5/9 |
| NH | 3/27 | 6/16 | 3/16 | TBD | 3/16 | TBD | 3/16 | 6/29 | 3/28 | 5/11 |
| NJ | 3/21 | 6/9 | 3/18 | TBD | 3/16 | TBD | 3/16 | TBD | 3/21 | 5/2 |
| NM | — | — | 3/13 | TBD | 3/12 | TBD | 3/16 | TBD | 3/24 | 5/15 |
| NY | 3/22 | 6/8 | 3/18 | TBD | 3/12 | TBD | 3/16 | TBD | 3/22 | 6/8 |
| NC | 3/30 | 5/8 | 3/14 | TBD | 3/14 | TBD | 3/17 | TBD | 3/30 | 5/8 |
| ND | — | — | 3/16 | TBD | — | — | 3/20 | TBD | — | — |
| OH | 3/23 | 5/20 | 3/16 | TBD | 3/12 | TBD | 3/15 | TBD | 3/23 | 5/4 |
| OK | — | — | 3/17 | TBD | 3/24 | 5/24 | 4/1 | 6/1 | 4/1 | 4/24 |
| OR | 3/23 | 6/19 | 3/16 | TBD | 3/12 | TBD | 3/17 | TBD | — | — |
| PA | 4/1 | 6/5 | 3/17 | TBD | 4/1 | 9/14 | 3/18 | 7/3 | 3/23 | 5/8 |
| RI | 3/28 | 5/9 | 3/16 | TBD | 3/17 | TBD | 3/17 | TBD | — | — |
| SC | 4/7 | 5/4 | 3/16 | TBD | 3/18 | TBD | 3/18 | 8/3 | — | — |
| SD | — | — | 3/16 | TBD | 4/6 | 4/28 | — | — | — | — |
| TN | 4/2 | 5/26 | 3/20 | TBD | 3/23 | TBD | 3/23 | TBD | 4/1 | 5/26 |
| TX | 4/2 | 5/1 | 3/19 | TBD | 3/21 | 6/4 | 3/21 | TBD | — | — |
| UT | — | — | 3/16 | TBD | 3/19 | 5/1 | 3/19 | TBD | — | — |
| VT | 3/24 | 5/15 | 3/18 | TBD | 3/13 | TBD | 3/17 | TBD | 3/25 | 5/4 |
| VA | 3/30 | 6/5 | 3/16 | TBD | 3/15 | TBD | 3/17 | TBD | 3/24 | 5/15 |
| WA | 3/23 | 7/3 | 3/13 | TBD | 3/11 | TBD | 3/16 | TBD | 3/25 | 7/3 |
| WV | 3/25 | 5/4 | 3/14 | TBD | 3/24 | TBD | 3/18 | TBD | 3/24 | 5/4 |
| WI | 3/25 | 5/13 | 3/18 | TBD | 3/17 | TBD | 3/17 | TBD | 3/25 | 5/11 |
| WY | — | — | 3/19 | TBD | 3/20 | TBD | 3/19 | 5/15 | — | — |

*Source:* [1]

*Note:* State policy information as of October 29, 2020. “TBD” indicates that a state suppression policy has not been lifted as of IHME’s October 29, 2020 update [1]. Missing values, indicated by a dash, indicate that a state never enforced a particular suppression policy.

**Table S2.2. Number of days during which both stay-at-home and nonessential business closures were enforced, weighted by each state’s share of U.S. GDP.**

| **State** | **GDP, 2019Q4**  **(in millions of dollars)** | **Percent of GDP** | **First day both orders enforced** | **Last day both orders enforced** | **Number of Days** | **Expected Number of Days** |
| --- | --- | --- | --- | --- | --- | --- |
| AL | $234,054 | 1.1% | 4/4 | 4/30 | 26 | 0 |
| AK | $55,759 | 0.3% | 3/28 | 4/24 | 27 | 0 |
| AZ | $372,522 | 1.7% | **—** | **—** | 0 | 0 |
| AR | $135,225 | 0.6% | **—** | **—** | 0 | 0 |
| CA | $3,183,251 | 14.7% | 3/19 | 8/1 | 135 | 20 |
| CO | $396,367 | 1.8% | 3/26 | 5/9 | 44 | 1 |
| CT | $288,985 | 1.3% | **—** | **—** | 0 | 0 |
| DE | $76,410 | 0.4% | 3/24 | 5/8 | 45 | 0 |
| DC | $148,231 | 0.7% | 3/30 | 5/29 | 60 | 0 |
| FL | $1,111,378 | 5.1% | **—** | **—** | 0 | 0 |
| GA | $625,329 | 2.9% | **—** | **—** | 0 | 0 |
| HI | $98,536 | 0.5% | 3/25 | 5/1 | 37 | 0 |
| ID | $82,265 | 0.4% | 3/25 | 5/1 | 37 | 0 |
| IL | $908,913 | 4.2% | 3/21 | 5/1 | 41 | 2 |
| IN | $381,733 | 1.8% | 3/25 | 5/18 | 54 | 1 |
| IA | $197,172 | 0.9% | **—** | **—** | 0 | 0 |
| KS | $175,703 | 0.8% | **—** | **—** | 0 | 0 |
| KY | $217,564 | 1.0% | **—** | **—** | 0 | 0 |
| LA | $267,051 | 1.2% | 3/23 | 5/1 | 39 | 0 |
| ME | $68,441 | 0.3% | 4/2 | 5/1 | 29 | 0 |
| MD | $434,312 | 2.0% | 3/30 | 5/15 | 46 | 1 |
| MA | $604,208 | 2.8% | **—** | **—** | 0 | 0 |
| MI | $548,567 | 2.5% | 3/24 | 5/7 | 44 | 1 |
| MN | $385,907 | 1.8% | **—** | **—** | 0 | 0 |
| MS | $120,429 | 0.6% | 4/3 | 4/27 | 24 | 0 |
| MO | $336,816 | 1.6% | **—** | **—** | 0 | 0 |
| MT | $52,948 | 0.2% | 3/26 | 4/26 | 31 | 0 |
| NE | $129,098 | 0.6% | **—** | **—** | 0 | 0 |
| NV | $180,406 | 0.8% | 3/31 | 5/9 | 39 | 0 |
| NH | $89,836 | 0.4% | 3/28 | 5/11 | 44 | 0 |
| NJ | $652,412 | 3.0% | 3/21 | 5/2 | 42 | 1 |
| NM | $105,263 | 0.5% | **—** | **—** | 0 | 0 |
| NY | $1,751,674 | 8.1% | 3/22 | 6/8 | 78 | 6 |
| NC | $596,383 | 2.8% | 3/30 | 5/8 | 39 | 1 |
| ND | $57,400 | 0.3% | **—** | **—** | 0 | 0 |
| OH | $706,764 | 3.3% | 3/23 | 5/4 | 42 | 1 |
| OK | $207,381 | 1.0% | **—** | **—** | 0 | 0 |
| OR | $255,418 | 1.2% | **—** | **—** | 0 | 0 |
| PA | $824,603 | 3.8% | 4/1 | 5/8 | 37 | 1 |
| RI | $64,441 | 0.3% | **—** | **—** | 0 | 0 |
| SC | $249,958 | 1.2% | **—** | **—** | 0 | 0 |
| SD | $54,057 | 0.3% | **—** | **—** | 0 | 0 |
| TN | $385,741 | 1.8% | 4/2 | 5/26 | 54 | 1 |
| TX | $1,918,065 | 8.9% | **—** | **—** | 0 | 0 |
| UT | $192,013 | 0.9% | **—** | **—** | 0 | 0 |
| VT | $35,271 | 0.2% | 3/25 | 5/4 | 40 | 0 |
| VA | $561,846 | 2.6% | 3/30 | 5/15 | 46 | 1 |
| WA | $610,488 | 2.8% | 3/25 | 7/3 | 100 | 3 |
| WV | $78,507 | 0.4% | 3/25 | 5/4 | 40 | 0 |
| WI | $351,922 | 1.6% | 3/25 | 5/11 | 47 | 1 |
| WY | $39,794 | 0.2% | **—** | **—** | 0 | 0 |
| **Total** | **$21,606,817** | **100.0%** | **—** | **—** | **—** | **42** |

*Sources:* [1, 34]; authors’ calculations.

*Note:* We set the number of days of suppression policies equal to zero for the 22 states that did not enforce both a nonessential business closure and stay-at-home order. Refer to Table S2.1 for the dates on which suppression policies were enacted and lifted in the U.S. states and the District of Columbia.

**Table S2.3. Number of days during which either a stay-at-home order or nonessential business closure order (inclusive) was enforced, weighted by each state’s share of U.S. GDP.**

| **State** | **GDP, 2019Q4**  **(in millions of dollars)** | **Percent of GDP** | **First day either order enforced** | **Last day either order enforced** | **Number of Days** | **Expected Number of Days** |
| --- | --- | --- | --- | --- | --- | --- |
| AL | $234,054 | 1.1% | 3/28 | 4/30 | 33 | 0 |
| AK | $55,759 | 0.3% | 3/28 | 4/24 | 27 | 0 |
| AZ | $372,522 | 1.7% | 3/30 | 5/16 | 47 | 1 |
| AR | $135,225 | 0.6% | **—** | **—** | 0 | 0 |
| CA | $3,183,251 | 14.7% | 3/19 | 8/1 | 135 | 20 |
| CO | $396,367 | 1.8% | 3/26 | 5/9 | 44 | 1 |
| CT | $288,985 | 1.3% | 3/23 | 5/20 | 58 | 1 |
| DE | $76,410 | 0.4% | 3/24 | 6/1 | 69 | 0 |
| DC | $148,231 | 0.7% | 3/25 | 5/29 | 65 | 0 |
| FL | $1,111,378 | 5.1% | 4/3 | 5/18 | 45 | 2 |
| GA | $625,329 | 2.9% | 4/3 | 8/1 | 120 | 3 |
| HI | $98,536 | 0.5% | 3/25 | 6/10 | 77 | 0 |
| ID | $82,265 | 0.4% | 3/25 | 5/1 | 37 | 0 |
| IL | $908,913 | 4.2% | 3/21 | 5/29 | 69 | 3 |
| IN | $381,733 | 1.8% | 3/24 | 5/18 | 55 | 1 |
| IA | $197,172 | 0.9% | 3/17 | 5/8 | 52 | 0 |
| KS | $175,703 | 0.8% | 3/30 | 5/4 | 35 | 0 |
| KY | $217,564 | 1.0% | 3/26 | 5/11 | 46 | 0 |
| LA | $267,051 | 1.2% | 3/22 | 5/15 | 54 | 1 |
| ME | $68,441 | 0.3% | 3/25 | 5/31 | 67 | 0 |
| MD | $434,312 | 2.0% | 3/23 | 5/15 | 53 | 1 |
| MA | $604,208 | 2.8% | 3/24 | 5/18 | 55 | 2 |
| MI | $548,567 | 2.5% | 3/23 | 6/1 | 70 | 2 |
| MN | $385,907 | 1.8% | 3/28 | 5/18 | 51 | 1 |
| MS | $120,429 | 0.6% | 4/3 | 4/27 | 24 | 0 |
| MO | $336,816 | 1.6% | 4/6 | 5/15 | 39 | 1 |
| MT | $52,948 | 0.2% | 3/26 | 5/1 | 36 | 0 |
| NE | $129,098 | 0.6% | **—** | **—** | 0 | 0 |
| NV | $180,406 | 0.8% | 3/21 | 5/9 | 49 | 0 |
| NH | $89,836 | 0.4% | 3/27 | 6/16 | 81 | 0 |
| NJ | $652,412 | 3.0% | 3/21 | 6/9 | 80 | 2 |
| NM | $105,263 | 0.5% | 3/24 | 5/15 | 52 | 0 |
| NY | $1,751,674 | 8.1% | 3/22 | 6/8 | 78 | 6 |
| NC | $596,383 | 2.8% | 3/30 | 5/8 | 39 | 1 |
| ND | $57,400 | 0.3% | **—** | **—** | 0 | 0 |
| OH | $706,764 | 3.3% | 3/23 | 5/20 | 58 | 2 |
| OK | $207,381 | 1.0% | 4/1 | 4/24 | 23 | 0 |
| OR | $255,418 | 1.2% | 3/23 | 6/19 | 88 | 1 |
| PA | $824,603 | 3.8% | 3/23 | 6/5 | 74 | 3 |
| RI | $64,441 | 0.3% | 3/28 | 5/9 | 42 | 0 |
| SC | $249,958 | 1.2% | 4/7 | 5/4 | 27 | 0 |
| SD | $54,057 | 0.3% | **—** | **—** | 0 | 0 |
| TN | $385,741 | 1.8% | 4/1 | 5/26 | 55 | 1 |
| TX | $1,918,065 | 8.9% | 4/2 | 5/1 | 29 | 3 |
| UT | $192,013 | 0.9% | **—** | **—** | 0 | 0 |
| VT | $35,271 | 0.2% | 3/24 | 5/15 | 52 | 0 |
| VA | $561,846 | 2.6% | 3/24 | 6/5 | 73 | 2 |
| WA | $610,488 | 2.8% | 3/23 | 7/3 | 102 | 3 |
| WV | $78,507 | 0.4% | 3/24 | 5/4 | 41 | 0 |
| WI | $351,922 | 1.6% | 3/25 | 5/13 | 49 | 1 |
| WY | $39,794 | 0.2% | **—** | **—** | 0 | 0 |
| **Total** | **$21,606,817** | **100.0%** | **—** | **—** | **—** | **65** |

*Sources:* [1, 34]; authors’ calculations.

*Note:* We set the number of days of suppression policies equal to zero for the 6 states that did not enforce either nonessential business closures or a stay-at-home order. Refer to Table S2.1 for the dates on which suppression policies were enacted and lifted in the U.S. states and the District of Columbia.
